# Reflex Xpert MTB/XDR Testing of Residual Rifampicin-Resistant Specimens: A Clinical Laboratory-Based Diagnostic Accuracy and Feasibility Study in South Africa

**DOI:** 10.1093/ofid/ofae437

**Published:** 2024-07-31

**Authors:** C M Centner, R Munir, E Tagliani, F Rieß, P Brown, C Hayes, T Dolby, W Zemanay, D M Cirillo, A David, S G Schumacher, C M Denkinger, M Ruhwald, V N Leukes, M P Nicol, I Van der Walt, G Kisten, M Gumede, A Mace, A Brink, W Stevens, L Scott, A Penn-Nicholson, H Cox, TB-CAPT Consortium, Vinzeigh Leukes, Adam Penn-Nicholson, Morten Ruhwald, Berra Erkosar, Samuel G Schumacher, Sunita Singh, Bernard Kivuma, Muhuminu Nuru, Judith Mlenge, Neema Shija, Deogratias Bulime, Dorcas Mnzava, Petro Sabuni, Hosiana Temba, Jamali Siru, Jerry Hella, Jonathan Msafiri, Maja Weisser, Mohamed Mbaruku, Mohamed Sasamalo, Alice Leonard, Ambilikile Malango, Annastazia Alexander, Faith Komakoma, Gloria Msigala, Kasmir Johaness, Grace Mhalu, Mwajabu Hamis, Priscilla Mlay, Robert Ndege, Sera Barasa, Swalehe Masoud, Theonestina Byakuzana, Anange Lwilla, Benedict Kayombo, Chacha Mangu, Christina Manyama, Theodora Mbunda, Elimina Siyame, Issa Sabi, Last Mwaipopo, Nyanda Elias Ntinginya, Raphael Edom, Willyhelmina Olomi, Delio Elisio, Dinis Nguenha, Edson Mambuque, Joaquim Cossa, Marta Cossa, Neide Gomes, Patricia Manjate, Shilzia Munguambe, Sozinho Acacio, Belen Saavedra, Helio Chiconela, Katia Ribeiro, António Machiana, Bindiya Meggi, Candido Azize Junior, Carla Madeira, Celso Khosa, Claudio Bila, Denise Floripes, Diosdélio Malamule, Sofia Viegas, Belén Saavedra, Carole Amroune, Joanna Ehrlich, Laura de la Torre Pérez, Sergi Sanz, Albero Garcia-Basteiro, Friedrich Riess, Sarah Mutuku, Tejaswi Appalarowthu, Leyla Larson, Katharina Kranzer, Michael Hoelscher, Norbert Heinrich, Maria del Mar Castro Noriega, Claudia M Denkinger, Saima Arif, Daniela Maria Cirillo, Elisa Tagliani, Federico Di Marco, Virginia Batignani, Akash Malhotra, David Dowdy, Claudia Schacht, Julia Buech, Caroline Stöhr, Marguerite Massinga Loembé, Pascale Ondoa, Nqobile Ndlovu, Fumbani Brown, Yonas Ghebrekristos, Cindy Hayes, Ilse Van der Walt, Shareef Abrahams, Puleng Marokane, Mbuti Radebe, Neil Martinson, Anura David, Lesley Scott, Lucky Ngwenya, Pedro Da Silva, Reyhana Solomon, Wendy Stevens, Charles Abongomera, Klaus Reither, Leon Stieger, Adrian Brink, Chad M Centner, Helen Cox, Judi van Heerden, Mark P Nicol, Nchimunya Hapeela, Parveen Brown, Reyhana Solomon, Widaad Zemanay, Tania Dolby

**Affiliations:** Division of Medical Microbiology, Department of Pathology, University of Cape Town, Cape Town, South Africa; National Health Laboratory Service, Groote Schuur Hospital, Cape Town, South Africa; Wits Diagnostic Innovation Hub, School of Pathology, Faculty of Health Sciences, University of the Witwatersrand, Johannesburg, South Africa; Emerging Bacterial Pathogens Unit, IRCCS Ospedale San Raffaele, Milan, Italy; Division of Infectious Diseases and Tropical Medicine, LMU University Hospital, LMU Munich, Munich, Germany; Division of Medical Microbiology, Department of Pathology, University of Cape Town, Cape Town, South Africa; National Health Laboratory Service, Gqeberha, South Africa; National Health Laboratory Service, Cape Town, South Africa; Division of Medical Microbiology, Department of Pathology, University of Cape Town, Cape Town, South Africa; Emerging Bacterial Pathogens Unit, IRCCS Ospedale San Raffaele, Milan, Italy; Wits Diagnostic Innovation Hub, School of Pathology, Faculty of Health Sciences, University of the Witwatersrand, Johannesburg, South Africa; Tuberculosis Programme, FIND, Geneva, Switzerland; Tuberculosis Programme, FIND, Geneva, Switzerland; Division of Infectious Disease and Tropical Medicine, Center for Infectious Diseases, Heidelberg University Hospital, and German Center for Infection Research (DZIF), Partner Site Heidelberg, Heidelberg, Germany; Tuberculosis Programme, FIND, Geneva, Switzerland; Tuberculosis Programme, FIND, Geneva, Switzerland; Marshall Centre for Infectious Diseases Research and Training, School of Biomedical Sciences, University of Western Australia, Perth, Western Australia, Australia; National Health Laboratory Service, Gqeberha, South Africa; National Health Laboratory Service, Cape Town, South Africa; National Health Laboratory Service, Cape Town, South Africa; Tuberculosis Programme, FIND, Geneva, Switzerland; Division of Medical Microbiology, Department of Pathology, University of Cape Town, Cape Town, South Africa; National Health Laboratory Service, Groote Schuur Hospital, Cape Town, South Africa; Welcome Centre for Infectious Disease Research in Africa and Institute of Infectious Disease and Molecular Medicine, University of Cape Town, Cape Town, South Africa; Wits Diagnostic Innovation Hub, School of Pathology, Faculty of Health Sciences, University of the Witwatersrand, Johannesburg, South Africa; National Priority Program, National Health Laboratory Service, Johannesburg, South Africa; Wits Diagnostic Innovation Hub, School of Pathology, Faculty of Health Sciences, University of the Witwatersrand, Johannesburg, South Africa; National Priority Program, National Health Laboratory Service, Johannesburg, South Africa; Tuberculosis Programme, FIND, Geneva, Switzerland; Division of Medical Microbiology, Department of Pathology, University of Cape Town, Cape Town, South Africa; Welcome Centre for Infectious Disease Research in Africa and Institute of Infectious Disease and Molecular Medicine, University of Cape Town, Cape Town, South Africa

**Keywords:** diagnostic accuracy, drug resistance, feasibility, tuberculosis, Xpert MTB/XDR

## Abstract

**Background:**

The World Health Organization–approved Xpert MTB/XDR test detects *Mycobacterium tuberculosis* and resistance to isoniazid, fluoroquinolones, ethionamide, and injectable drugs directly in specimens. This pragmatic, laboratory-based study assessed the diagnostic accuracy and feasibility of a reflex testing approach, where Xpert MTB/XDR was performed on residual specimens previously processed for Xpert MTB/RIF Ultra.

**Methods:**

Routine respiratory specimens, processed for Xpert MTB/RIF Ultra, were stored in sample reagent buffer at 2°C–8°C. If rifampicin resistant, the residual specimen was assessed for adequate volume (≥2 mL) and tested with Xpert MTB/XDR, with storage time recorded. A second specimen was used for routine and reference standard testing (culture and sequencing).

**Results:**

Specimens (99% sputum) from 763 participants submitted to 2 large routine laboratories were included. Xpert MTB/XDR yielded valid resistance detection results in 639 (84%), compared with 507 (66%) for routine testing (difference [95% CI], 18% [13%–22%]). The median turnaround time for results was 23 hours for Xpert MTB/XDR and 15 days for routine testing. While 748 specimens (98%) were ≥2 mL, only 102 (13%) were stored for ≤4 hours. By the reference standard, 284 of 394 (72%) were isoniazid resistant, and 57 of 380 (15%) were fluroquinolone resistant. The sensitivities of Xpert MTB/XDR were 94% (95% CI, 91%–97%) for isoniazid and 91% (81%–97%) for fluoroquinolone resistance detection. The specificities were 98% (94%–100%) and 100% (98%–100%), respectively.

**Conclusions:**

Xpert MTB/XDR performed favorably compared with the reference, and the reflex testing approach increased results availability over routine testing, while dramatically decreasing turnaround time from weeks to hours. Laboratory workflow precluded testing within the manufacturer-recommended 4-hour storage time, but longer storage did not appear detrimental.

## Teaser

The Xpert MTB/XDR assay performed favorably for the detection of isoniazid, fluoroquinolones, and injectable tuberculosis drug resistance in a reflex testing algorithm that dramatically reduced turnaround time in 2 high-throughput clinical laboratories.

## Graphical Abstract

**Graphical Abstract:**
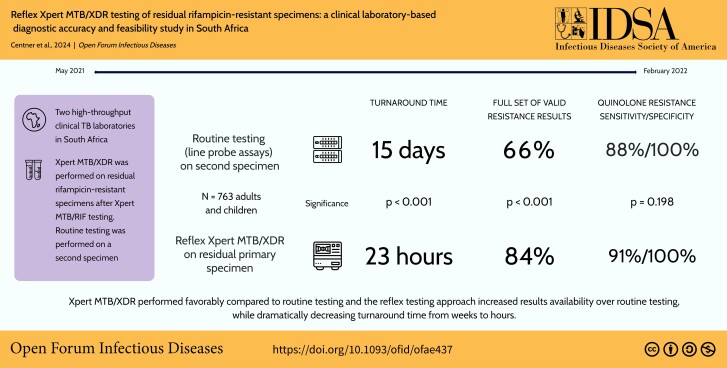
https://tidbitapp.io/tidbits/reflex-xpert-mtb-xdr-testing-of-residual-rifampicin-resistant-specimens-a-clinical-laboratory-based-diagnostic-accuracy-and-feasibility-study-in-south-africa

Globally, the largest barrier to providing effective treatment for the 410 000 individuals estimated to develop multidrug (MDR) or rifampicin-resistant (RR) tuberculosis annually is rapid and effective diagnosis; only 2 in 5 have rifampicin resistance detected and receive any second-line tuberculosis treatment [1]. In addition, providing the most effective second-line treatment for MDR/RR tuberculosis and preventing emergence of further drug resistance requires knowledge of susceptibility to isoniazid and key second-line tuberculosis drugs included in currently recommended regimens [2].

South Africa has a high burden of MDR/RR tuberculosis, with 11 000 individuals estimated to develop MDR/RR tuberculosis in 2022 and approximately 7000 of these diagnosed [1]. The World Health Organization (WHO)–endorsed Xpert MTB/RIF and Xpert MTB/RIF Ultra (Cepheid) low-complexity automated nucleic acid amplification tests have been rolled out widely in South Africa and elsewhere, providing rapid detection of *Mycobacterium tuberculosis* (*Mtb*) complex as well as detection of rifampicin resistance mutation for all individuals investigated for tuberculosis [3]. However, current testing for resistance to isoniazid and second-line tuberculosis drugs has relied on line probe assays (LPAs) and phenotypic drug susceptibility testing (DST), resulting in incomplete results and delays in diagnosis [4].

In 2021, WHO endorsed the Xpert MTB/XDR (Cepheid) assay to detect resistance to isoniazid, fluoroquinolones, ethionamide, and second-line injectable tuberculosis drugs [5]. This rapid, cartridge-based real-time PCR assay, which runs on a 10-color version of the same platform as Xpert MTB/RIF Ultra, provides results within 2 hours, directly in primary specimens [6, 7], thereby simplifying processing compared with other WHO-endorsed molecular assays, such as LPAs. The Genotype MTBDR*plus* and MTBDR*sl* (Bruker) are technically complex and perform better in cultured isolates, especially in those with sputum smear–negative tuberculosis [8, 9]. Available diagnostic accuracy data for the Xpert MTB/XDR assay suggest high sensitivity for isoniazid (94.2%) and for fluoroquinolones (93.2%) against phenotypic susceptibility testing, along with high sensitivity for ethionamide testing (98.0%) and moderate sensitivity for amikacin (86.1%) against genotypic resistance testing [10].

While next-generation sequencing approaches have the potential to provide more complete drug resistance data, including for the newer tuberculosis drugs, there are currently costs and complexities that may limit widespread implementation [11]. Therefore, rapid, scalable and affordable susceptibility tests for both first- and second-line tuberculosis drugs, such as the Xpert MTB/XDR assay, require further evaluation, both for diagnostic performance and to support different implementation approaches across different settings. This laboratory-based study, conducted within 2 large routine clinical laboratories in South Africa, evaluated Xpert MTB/XDR for diagnostic accuracy and investigated the feasibility of a reflex testing strategy in which both Xpert MTB/RIF Ultra and Xpert MTB/XDR were performed on the same specimen.

## METHODS

### Study Design and Setting

This prospective noninterventional laboratory-based diagnostic accuracy and observational study included routine clinical respiratory specimens (with no age restriction) submitted to 2 high-throughput routine clinical laboratories in South Africa: site 1 was the South African National Health Laboratory Service tuberculosis laboratory in Green Point, Cape Town, Western Cape, and site 2 was the National Health Laboratory Service tuberculosis laboratory in Gqeberha, Eastern Cape. Both sites provide diagnostic services to primary, secondary, and tertiary public sector healthcare facilities in their respective areas and process approximately 60 000 and 40 000 specimens for tuberculosis diagnosis per month, respectively. The study was approved by the National Health Laboratory Service (PR2010407), University of Cape Town Human Research Ethics Committee (reference 607-2020), and the University of the Witwatersrand Human Research Ethics Committee (reference M1911201). The study was an arm of the TB-CAPT trial (Close the gap, increase Access, Provide adequate Therapy; https://www.tb-capt.org/; clinicaltrials.gov NCT04567368).

Based on a conservative estimate of 61% prevalence for isoniazid-resistance and 11% for fluoroquinolone-resistance among patients with RR tuberculosis across both settings [12], and Xpert MTB/XDR sensitivity and specificity estimates for individual drug targets at 70% and 90%, respectively, a 95% Pearson-Clopper confidence interval (CI) width range of 4.7%–30% required a minimal sample size of 320 participants for calculation of sensitivity and specificity for fluoroquinolones. Accounting for anticipated loss-to-follow-up, negative and contaminated cultures, and failure of reference standard testing, a total of approximately 750 specimens was estimated to be required from both sites.

### Patient Consent Statement

Individual informed consent was waived by both ethical review committees, as only residual specimens were used in this study.

### Xpert MTB/RIF Ultra and Xpert MTB/XDR

**Figure 1.**
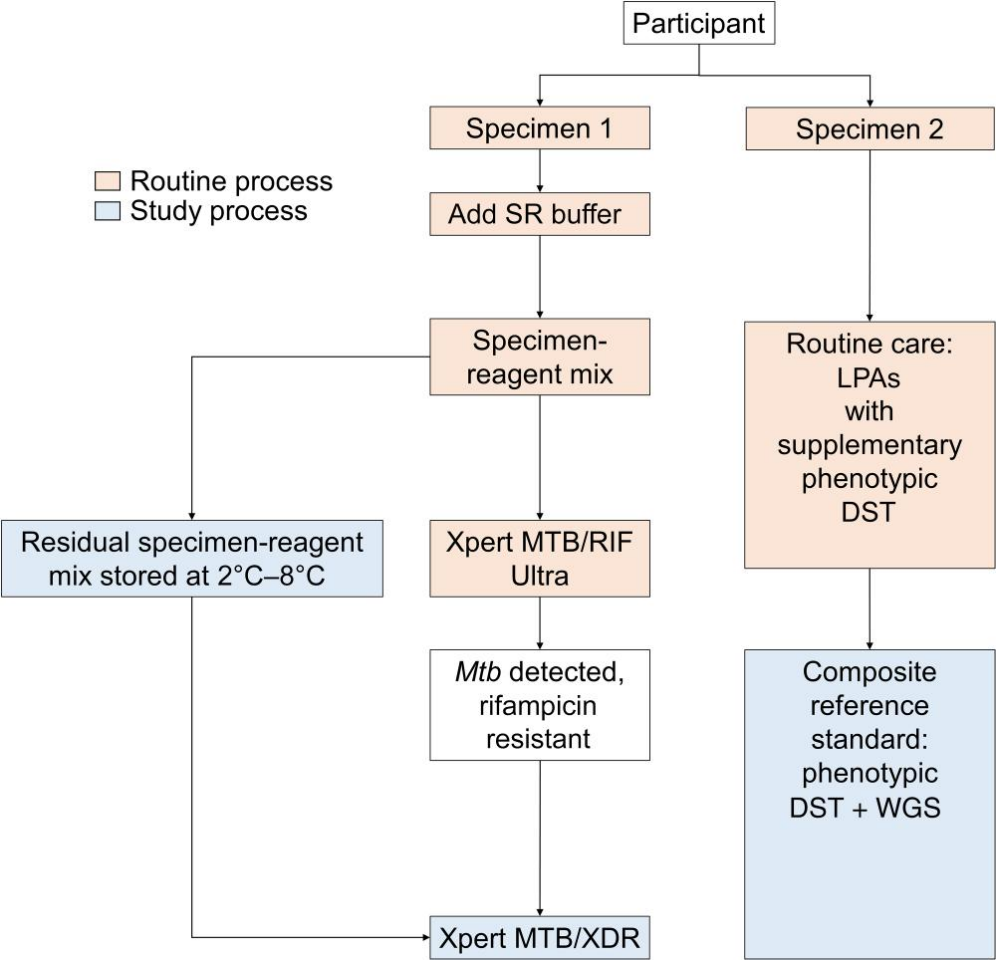
Specimen flow in the routine laboratory and in the study. Abbreviations: DST, drug susceptibility testing; LPAs, line probe assays; *Mtb*, *Mycobacterium tuberculosis*; SR, Xpert Ultra Sample Reagent; WGS, whole-genome sequencing.

Consecutive respiratory specimens submitted to the laboratory were routinely processed by the addition of Xpert Ultra Sample Reagent (SR) buffer in a 2:1 ratio to yield the “specimen-reagent mix.” A 2 -mL aliquot of specimen-reagent mix was then submitted for Xpert Ultra and the residual mix stored at 2ºC–8ºC (Figure 1). After completion of testing, the mix from specimens found to be resistant to rifampicin was retrieved, and 2 mL was submitted for Xpert MTB/XDR without additional processing. Any aliquots of residual mix <2 mL were topped up to 2 mL with SR buffer. The period between processing for Xpert Ultra and start of the Xpert MTB/XDR assay run was noted (“storage time”). In this way, both Xpert Ultra and Xpert MTB/XDR were performed on the same specimen (specimen 1). Xpert MTB/XDR results were not made available for routine clinical management.

### Routine Testing

In addition to Xpert Ultra testing on specimen 1, routine diagnostics for second-line drug-susceptibility testing (in participants with RR tuberculosis identified) included a second specimen (specimen 2) that underwent molecular testing using MTBDR*plus* to detect mutations predictive of rifampicin and isoniazid resistance and MTBDR*sl* for fluoroquinolones and injectables. These LPAs were performed either on the primary specimen or on a cultured BACTEC MGIT isolate (Becton, Dickinson & Co) in cases where primary LPA failed or was indeterminate, with supplemental confirmatory BACTEC MGIT phenotypic susceptibility testing per the national algorithm (confirmatory phenotypic DST for all isoniazid-susceptible and fluoroquinolone-resistant specimens). In a predefined secondary analysis, routine resistance testing (MTBDR*plus* and MTBDR*sl* results only) was compared with Xpert MTB/XDR. The 2 sites followed different tuberculosis testing algorithms. At site 1, specimen 2 was submitted at the same time as specimen 1. At site 2, specimen 2 was submitted when patients returned to receive the RR tuberculosis result.

### Reference Standard Testing

Reference standard phenotypic DST was also performed on specimen 2 using BACTEC MGIT (Becton, Dickinson & Co). The critical concentration was 0.1 mg/L for isoniazid, 1.0 mg/L for levofloxacin, and 2.5 mg/L for kanamycin. No phenotypic DST was performed for ethionamide.

For whole-genome sequencing (WGS), an aliquot of cording acid-fast bacilli-positive mycobacterial growth indicator tube material from specimen 2 was heat inactivated. Nucleic acid extraction and purification was performed using a Maxwell 16 FFPE Tissue LEV DNA Purification Kit (Promega). Paired-end libraries were constructed using the Nextera XT DNA Library Prep Kit (Illumina) and sequenced on an Illumina NextSeq platform with the NextSeq High Output kit (300 cycles), aiming for a 50× sequencing depth. Sequencing data were analyzed using the MTBseq pipeline (version 1.0.2) to identify all variants at a 10% allele frequency threshold in the genomes and define *Mtb* complex lineage [13].

The composite reference standard (CRS) comprised phenotypic DST plus prediction of resistance to isoniazid, ethionamide, fluoroquinolones, and injectables by WGS. Isolates were defined as susceptible to a given drug if they tested susceptible by phenotypic DST and if no mutations predictive of resistance were identified by WGS. Isolates were defined as resistant if they tested resistant by phenotypic DST or if a mutation predictive of resistance was identified [14].

### Feasibility and Turnaround Time

The feasibility indicators evaluated were the proportion of participants with ≥2 mL of residual specimen-reagent mix remaining after Xpert Ultra (specimens requiring topping up were also tested with Xpert MTB/XDR), the proportion of stored specimen-reagent mixes tested within the manufacturer's recommended interval (≤4 hours), and the proportion of participants with valid resistance profiles (defined as the instrument or laboratory report showing either a susceptible or resistant result for all of isoniazid, fluoroquinolones, and injectables). Storage time was defined as the period between addition of SR buffer to the start of the MTB/XDR run. Turnaround time was defined as the period between registration of receipt of the specimen in the laboratory and the availability of a finalized report.

### Analysis

Proportions were compared using the χ^2^ test. The sensitivity and specificity of Xpert MTB/XDR and corresponding 95% CIs against the CRS were estimated using the Wilson score method. When comparing the sensitivity and specificity of Xpert MTB/XDR against the CRS with sensitivity and specificity against routine testing, CIs were calculated using the McNemar χ^2^ test. Turnaround time and storage time were expressed as median with interquartile range (IQR) and were compared between sites and between Xpert MTB/XDR and standard-of-care testing using the rank sum test. Differences were deemed statistically significant at *P* < .05.

## RESULTS

**Figure 2.**
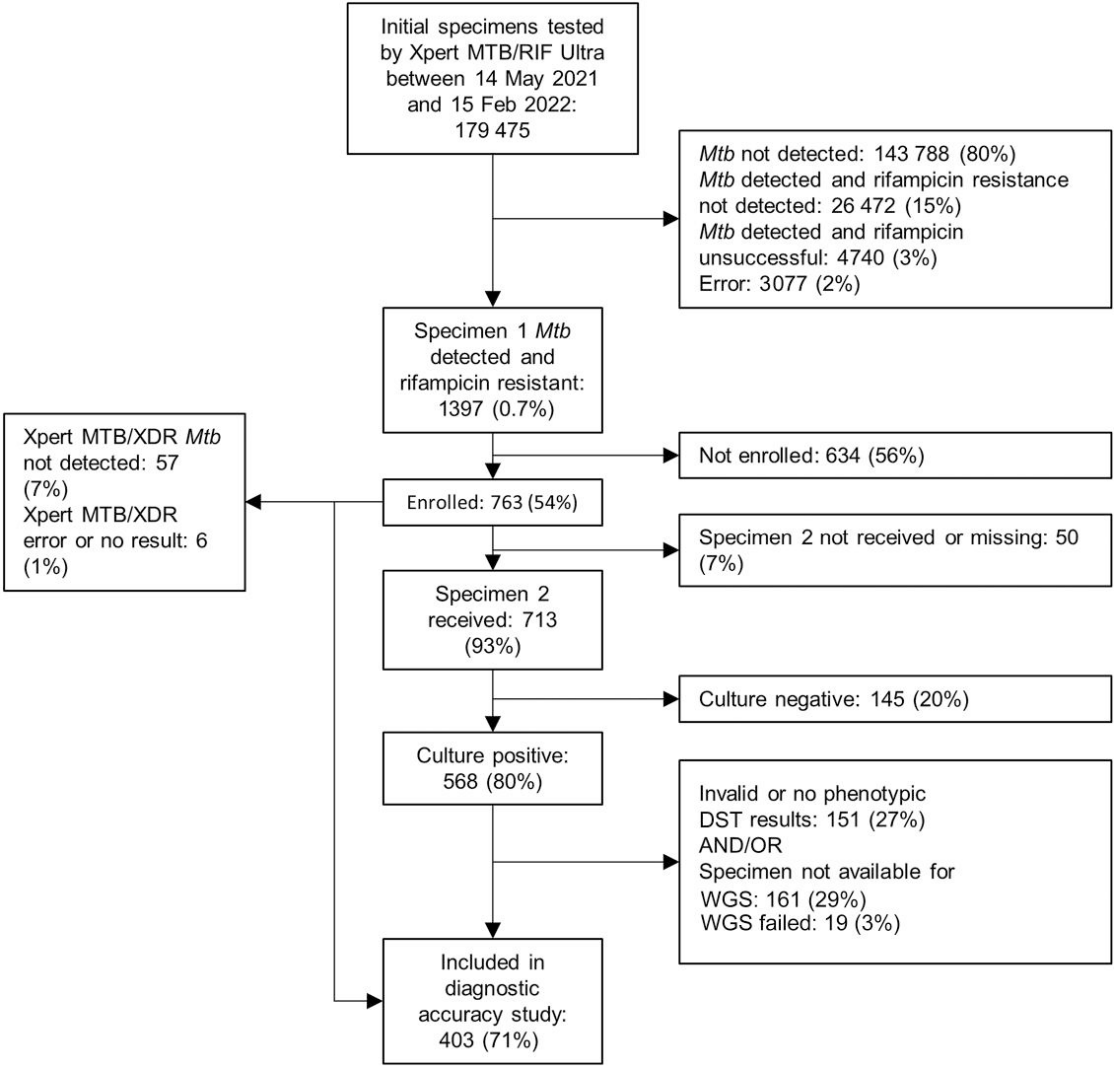
Study enrollment and retention flowchart. Reasons for nonenrollment included coronavirus disease 2019–related disruptions, intermittent power supply at both sites, staffing shortages, not all staff trained in study procedures, and periods of high clinical demand leading to technologists prioritizing routine clinical work over study procedures. Reasons for invalid or no phenotypic drug susceptibility testing (DST) results or specimens not available for whole-genome sequencing (WGS) included mycobacterial growth indicator tube not retrieved at the conclusion of standard-of-care testing or retained by the laboratory for operational reasons, repeated contamination on subculture, or nonviable isolate. The numbers of specimens included in the diagnostic accuracy study differed between drugs.

Specimens from 763 participants were included in the study between 14 May 2021 and 15 February 2022 (Figure 2): 372 (49%) from site 1 and 391 (51%) from site 2. The median age of participants (IQR) was 36.2 (29.3–44.9) years. Specimens from 31 children (4%) aged <18 years were included. Specimens were expectorated sputum in all but 3 of 763 (1 induced sputum, 1 tracheal aspirate and 1 bronchoalveolar lavage specimen). Specimens were submitted from primary care clinics (87%), secondary hospital wards (8%), tertiary hospital wards (4%), and hospital intensive care units (<1%). Overall, 713 participants (93%) had 2 specimens available for testing; 449 (63%) of these were submitted on the same day.

### Feasibility and Turnaround Time

Most residual specimen-reagent mix aliquots (748 [98%]) were ≥2 mL and therefore did not require top-up for Xpert MTB/XDR testing (Table 1). The proportion of specimens with storage time ≤4 hours, the limit defined by the Xpert MTB/XDR package insert, was 102 of 760 (13%) and differed substantially between sites 1 and 2 (27% vs 1%, respectively; difference in proportion [95% CI], 26% [21%–30%]; *P* < .001). *Mtb* was detected by Xpert MTB/XDR in 700 specimens (92%) and was not detected in 57 (7%). The remaining 6 specimens (1%) generated an error or “no result” output. Of the 57 Xpert MTB/XDR tests that did not detect *Mtb*, 52 (91%) were “very low” and the remaining 5 were “low” on Xpert Ultra semiquantitation. The *Mtb* detection rate on Xpert MTB/XDR was 314 of 327 (96%) in acid-fast bacilli smear-positive specimens and 386 of 436 (89%) in smear-negative specimens (difference in proportion [95% CI], 8% [4%–11%]; *P* < .001).

**Table 1.** Feasibility Outcomes, Xpert MTB/XDR *Mycobacterium* Tuberculosis Detection, Valid Resistance Detection Results, and Turnaround Time—Overall and by Site

Variable	Overall (N = 763)	Site 1 (N = 372)	Site 2 (N = 391)	Site 1 vs Site 2: Difference in Proportions (95% CI), %	*P* Value
Specimen-reagent mix					
Volume ≥2 mL remaining after Xpert Ultra testing, no. (%)	748 (98)	357 (96)	391 (100)	…	.38
Storage time, median (IQR), h	7.7 (4.7–14.9)^a^	4.8 (3.8–6.0)^b^	14.43 (11.6–19.4)^c^	…	<.001
Storage time ≤4 h, no. (%)	102 (13)^a^	99 (27)^b^	3 (1)^c^	26 (21–30)	<.001
Xpert MTB/XDR results, no. (%)					
*Mtb* detected	700 (92)	342 (92)	358 (92)	0 (−3 to 4)	.33
*Mtb* not detected	57 (7)	24 (6)	33 (8)	−2 (−10 to 5)	.33
Error	4 (0.5)	4 (1)	0 (0)	…	…
No result	2 (0.3)	2 (0.5)	0 (0)	…	…
Valid resistance detection, no. (%)^d^					
Xpert MTB/XDR^e^	639 (84)^f^	310 (83)^g^	329 (84)	−1 (−4 to 6)	.76
Routine testing	507 (66)	277 (74)	230 (59)	16 (9–22)	<.001
Xpert MTB/XDR vs routine testing					
Difference in proportions (95% CI), %	17 (13–22)	8 (3–14)	25 (19–31)	…	…
*P* value	<.001	<.001	<.001	…	…
Turnaround time					
Xpert MTB/XDR, median (IQR), h	23.1 (17.7–31.1)	17.7 (16.5–18.9)	30.5 (26.2–45.5)	…	<.001
Routine testing, median (IQR), h	362.3 (125.0–671.5)	139.6 (91.8–498.2)	597.5 (266.7–893.3)	…	<.001
Routine testing, median (IQR), d	15.1 (5.2–28.0)	5.8 (3.8–20.8)	24.9 (11.1–37.2)	…	<.001
*P* value (Xpert MTB/XDR vs routine testing)	<.001	<.001	<.001	…	…

Abbreviations: CI, confidence interval; IQR, interquartile range; *Mtb*, *Mycobacterium tuberculosis*.

^a^N = 760 (data missing for 3 participants).

^b^N = 371 (data missing for 1 participants).

^c^N = 389 (data missing for 2 participants).

^d^Valid resistance detection results defined as either resistance detected or not detected for all of isoniazid, fluoroquinolones, and injectables. Reasons for nonvalid Xpert MTB/XDR results included *Mtb* not detected, error, “no result,” and “resistance indeterminate” result. Reasons for nonvalid routine testing results included second specimen not received and line probe assay result indeterminate.

^e^Per protocol (*Mtb* negative specimens were not excluded).

^f^N = 759 (data missing for 4 participants).

^g^N = 370 (data missing for 4 participants).

A full set of valid resistance detection results for Xpert MTB/XDR was obtained in 639 specimens (84%), while routine testing generated a full set of valid susceptibility results in 507 (66%) (difference in proportion [95% CI], 18% [13%–22%]; *P* < .001). The main reason for not having a full set of valid routine susceptibility testing results was no second specimen received (141 of 256 [55%]), with the remaining reasons being a combination of uninterpretable results and culture negativity (data not shown).

Routine testing was done using direct first-line LPA in 343 (62%) and direct second-line LPA in 257 (50%); LPA was performed on the cultured isolate for the remaining specimens. The median turnaround time (IQR) for Xpert MTB/XDR was 23.1 (17.7–31.1) hours, compared with 15.1 (5.2–28.0) days for routine (LPA) testing. Comparing specimen mixes that were held for ≤4 hours with those held for >4 hours, there were no significant difference in the proportions of specimens with *Mtb* detected (89% vs 92%, respectively) or in proportions with valid resistance detection results (78% vs 85%; Table 2). There was also no significant difference using an 8-hour cutoff (84% vs 83%; *P* = .74).

**Table 2.** *Mycobacterium tuberculosis* Detection and Valid Resistance Detection Results Obtained by Xpert MTB/XDR With Specimen-Reagent Mixes Stored for ≤4 Versus >4 Hours After Processing for Xpert MTB/RIF Ultra

Xpert MTB/XDR Results	Isolates, No. (%)			Storage ≤4 vs >4 h: Difference in Proportions (95% CI), %	*P* Value (Storage Time ≤4 vs >4 h)
	Overall (n = 760)	Storage ≤4 h (n = 102)	Storage >4 h (n = 658)		
*Mtb* detected	697 (92)	91 (89)	606 (92)	−3 (−9 to 3)	.33
*Mtb* not detected	57 (8)	6 (6)	51 (8)	…	…
Error	4 (<1)	3 (3)	1 (<1)	…	…
No result	2 (<1)	2 (2)	0 (0)	…	…
Xpert MTB/XDR valid resistance detection^a^	636 (84)	80 (78)	556 (84)	−6 (−15 to 2)	.12

Abbreviations: CI, confidence interval; *Mtb*, *Mycobacterium tuberculosis.*

^a^Valid resistance detection results defined as either resistance detected or not detected for all drugs combined.

### Diagnostic Accuracy

Results of the CRS and Xpert MTB/XDR, including sensitivities, specificities, and positive and negative predictive values, are presented in Table 3. Reference standard testing on specimen 2 showed that 284 of 394 specimens (72%) were isoniazid and 57 of 380 (15%) were fluroquinolone resistant. The sensitivity of Xpert MTB/XDR for the detection of isoniazid resistance was 94%, and the specificity was 98%; for fluoroquinolones, the sensitivity was 91% and the specificity, 100%. There were no differences in sensitivity and specificity compared with the CRS between the Xpert MTB/XDR assay and routine (LPA) testing (Table 4).

**Table 3.** Proportions of Isolates Resistant to Isoniazid, Fluoroquinolones, Injectables, and Ethionamide With the Composite Reference Standard and Xpert MTB/XDR and Sensitivity, Specificity, and Positive and Negative Predictive Values for Xpert MTB/XDR^a^

Drug	Total No. of Isolates	Resistant Isolates, No. (%)		Value for Xpert MTB/XDR (95% CI), %			
		CRS	Xpert MTB/XDR	Sensitivity	Specificity	PPV	NPV
Isoniazid	394	284 (72)	269 (68)	94 (91–97)	98 (94–100)	99 (97–100)	86 (79–92)
Fluoroquinolones	380	57 (15)	53 (14)	91 (81–97)	100 (98–100)	98 (90–100)	99 (97–100)
Injectables	368	49 (13)	43 (12)	88 (75–95)	100 (99–100)	100 (92–100)	98 (96–99)
Ethionamide	395	184 (47)	173 (44)	91 (86–95)	97 (94–99)	97 (93–99)	92 (88–96)

Abbreviations: CI, confidence interval; CRS, composite reference standard; NPV, negative predictive value; PPV, positive predictive value.

^a^Sensitivity, specificity, NPV, and PPV were measured against the CRS. The CRS comprised isoniazid, levofloxacin, and kanamycin phenotypic drug susceptibility testing plus predicted resistance on whole-genome sequencing. Isolates were defined as susceptible to a given drug if they tested susceptible by both methods and resistant if they tested resistant by either method. Phenotypic susceptibility testing was not performed for ethionamide.

**Table 4.** Sensitivity and Specificity of Xpert MTB/XDR and Routine Testing^a^

Drug	Value (95% CI), %				*P* Value (Xpert MTB/XDR vs SOC Testing)
	Xpert MTB/XDR		Routine Testing		
	Sensitivity	Specificity	Sensitivity	Specificity	
Isoniazid	94 (91–97)	98 (94–100)	93 (90–96)	98 (94–100)	.63
Fluoroquinolones	91 (81–97)	100 (98–100)	88 (77–95)	100 (99–100)	.20
Injectables	88 (75–95)	100 (99–100)	89 (77–96)	99 (98–100)	.16

Abbreviations: CI, confidence interval; SOC, standard-of-care.

^a^Sensitivity and specificity measured against to the composite reference standard. Specimens were defined as resistant to a given drug if resistance was detected by line probe assay performed on either the primary specimen or the culture isolate. Results of routine confirmatory phenotypic susceptibility testing were excluded to avoid incorporation bias with the composite reference standard.

## DISCUSSION

This study demonstrated effective implementation of a reflex Xpert MTB/XDR testing strategy that does not rely on receipt of a second specimen for second-line drug resistance testing. As implemented in 2 large, high-throughput tuberculosis laboratories, the reflex testing strategy resulted in a substantially higher proportion of participants with valid drug resistance testing results compared with routine testing (84% vs 66%), results which, importantly, were available within hours rather than weeks. The reflex strategy required minimal deviations in workflow, namely the storage of residual processed specimens followed by retrieval of RR specimens after the Xpert MTB/RIF Ultra run. Most of these specimen-reagent mixes were suitable for further testing based on the volume of specimen remaining. While maintaining storage duration within manufacturer recommendation of ≤4 hours was problematic, there was no evidence that prolonged storage affected the yield of Xpert MTB/XDR. However, these data highlight the need for validation of longer storage times under well-defined conditions.

This testing approach has the potential to further reduce gaps in the diagnostic and treatment cascade for MDR/RR tuberculosis. Overall, 84% of participants had valid drug resistance results, compared with only 66% with routine testing. Given that the fluoroquinolones remain a key drug class in currently recommended MDR/RR tuberculosis treatment regimens, including in the newer BPaLM (bedaquiline, pretomanid, linezolid, and moxifloxacin) regimen, rapid drug resistance results would enable rapid initiation of alternative treatment regimens in the case of resistance to fluoroquinolones [2]. Given the significant emergence of resistance to bedaquiline [15], and limited data on the robustness of the BPaL regimen (without the inclusion of a fluoroquinolone) in preventing bedaquiline resistance acquisition, earlier access to fluoroquinolone DST may prevent further bedaquiline resistance emergence by facilitating strengthened treatment regimens. This is particularly relevant given the current lack of rapid DST methods for key drugs such as bedaquiline and linezolid.

In addition, while this study used the Xpert MTB/XDR assay only on residual RR tuberculosis specimens, a similar approach could be used for all specimens testing MTB positive. This would enable diagnosis of isoniazid-monoresistant tuberculosis, where cost-effective, and could further inform patient selection for newer 4-month regimens for drug-susceptible tuberculosis [16]. For all individuals with tuberculosis, including those with MDR/RR, isoniazid-monoresistant, and drug-susceptible tuberculosis, access to rapid DST that allows for earlier treatment initiation with more appropriate regimens has the potential to both lower mortality rates and reduce the risk of further resistance emergence, through the provision of more effective treatment regimens [17–19].

The predominant reason for incomplete routine test results was the requirement for a second specimen for culture and LPAs. In the current study, strategies for specimen collection varied between the 2 sites; site 1 required both specimens to be submitted up front (where possible), while site 2 required submission of a second specimen when the first specimen returned a positive *Mtb* result. These differences are reflected in the substantially longer turnaround time for routine testing at site 2 compared with site 1 (6 vs 25 days) and the lower proportion of participants with valid routine DST results and highlight the need to carefully evaluate different implementation approaches for new and existing tuberculosis diagnostics in different settings. However, given our continued reliance on phenotypic drug resistance testing or end-to-end targeted next-generation sequencing solutions for newer drugs, such as bedaquiline [11], a second specimen is likely still required.

The diagnostic accuracy data shown here add to the growing body of data demonstrating the accuracy of the Xpert MTB/XDR cartridge for detecting resistance to key drugs [10, 20]. Sensitivity and specificity were high for isoniazid, fluoroquinolones, and ethionamide. These results are similar to those described elsewhere, despite the novel strategy of using the residual specimen from Xpert Ultra testing, longer than recommended storage times and implementation in a routine laboratory. Discordance between DST results from different specimens and obtained using different DST methods is relatively common for MDR/RR tuberculosis [21, 22]. In this and other studies, discordance between Xpert MTB/XDR and reference standard results may have been due to different specimens “sampling” from different pulmonary tuberculous lesions or the presence of mixed infections or heteroresistance in the same specimen. While direct testing on specimens is valuable to obtain rapid results and inform patient care, there is additional value in conducting DST on cultured isolates, as use of sequencing approaches, including targeted sequencing, may detect underlying heteroresistance, provide more accurate susceptibility results for some drugs, and further inform individual regimen design.

The Xpert MDR/XDR cartridge includes testing for resistance to isoniazid, ethionamide and the second-line injectable tuberculosis drugs, in addition to fluoroquinolones. In many settings, a significant proportion of individuals with MDR/RR tuberculosis are infected with RR tuberculosis with isoniazid susceptibility (27% by Xpert MTB/XDR in this study) [23]. For these individuals, early knowledge of isoniazid susceptibility may enable inclusion of isoniazid in a second-line MDR/RR tuberculosis regimen. Indeed, South Africa is moving toward use of medium- and high-throughput rapid genotypic testing platforms that would enable concomitant rifampicin and isoniazid DST [24, 25]. While ethionamide remains a recommended drug for some patients with MDR/RR tuberculosis, the injectable drugs are no longer recommended due to significant toxicity [2]. Indeed, replacing the injectable drugs in second-line regimens was suggested more than a decade ago [26]. Further iterations of Xpert cartridges, and indeed other rapid molecular tests (including targeted sequencing approaches), should ideally include tests for currently used drugs, such as bedaquiline or linezolid and/or combining rifampicin, isoniazid, and fluoroquinolones.

This study provides data from a real-world, routine setting with the use of a robust reference standard. The study's execution within a demanding high-throughput environment by well-trained staff added further rigor. Conversely, it might be difficult to translate study findings to other contexts, particularly those without 24-hour service or access to refrigeration. Further limitations include potential variability due to the index specimen being different from that used for the reference standard and frequently having been collected at a different time. In addition, the absence of participant human immunodeficiency virus (HIV) status and the limited inclusion of extrapulmonary cases and pediatric specimens might affect the generalizability of the results.

While South Africa is now pursuing a strategy whereby Xpert MTB/RIF Ultra will be replaced with higher-throughput PCR testing strategies for high-volume laboratories [24, 25], reflex testing of specimens with rifampicin resistance using Xpert MTB/RIF Ultra remains a viable strategy in many countries with a high MDR/RR tuberculosis burden that have already rolled out Xpert platforms. Currently, only half of all individuals with diagnosed MDR/RR tuberculosis receive fluroquinolone resistance testing as a minimum [23]. In South Africa, Xpert MTB/XDR will supersede the use of 2 LPAs and is therefore likely to result in cost savings. Given South Africa's previous experience in leading global efforts to provide universal drug resistance testing and scaling up access to newer MDR/RR tuberculosis treatment regimens, current innovations offer an opportunity to further evaluate different testing strategies.

In conclusion, this study provides substantial evidence to support the feasibility and potential benefits of implementing reflex Xpert MTB/XDR testing in routine clinical laboratories. While challenges in maintaining optimal storage conditions and the need for further investigations remain, the findings strongly suggest improved diagnostic efficiency and potential benefits for patient care.
